# Sexuality Support After Spinal Cord Injury: What is Provided in Australian Practice Settings? Sexuality Support After Spinal Cord Injury

**DOI:** 10.1007/s11195-022-09756-w

**Published:** 2022-08-06

**Authors:** Chloe Bryant, Tammy Aplin, Jenny Setchell

**Affiliations:** 1 School of Health and Rehabilitation Sciences, The University of Queensland, Brisbane, Australia; 2 Allied Health Research Collaborative, The Prince Charles Hospital, Chermside, Australia

**Keywords:** Sexuality, Sexual health, Spinal cord injuries, Neurological rehabilitation, Delivery of health care, Australia

## Abstract

This study sought to understand what sexuality support Australian health professionals currently provide to people with spinal cord injury (SCI) and their perspectives on what changes may better support the sexuality needs of people with SCI. Australian Health professionals who had worked with people with SCI within the last 10 years were invited to participate in an online survey. Results were analyzed using content analysis and descriptive statistics. The 39 participants were from a range of health professions including medical, allied health, nursing, and peer support. Participants worked in various service settings, with the highest frequency in the community (33%) or inpatient rehabilitation (28%). Analysis indicated 85% of participants had provided sexuality support, however this provision was rarely routine. Discussing sexuality education topics were reported to be routinely provided for less than 16% of participants. Overall, 32% of participants felt sexuality was addressed ‘not well at all’ in their workplace. Qualitative analysis of open-ended responses produced five themes: *barriers to supporting sexuality, health professionals require training, utilizing a team approach, responsibility to initiate conversation*, and *involving others in support.* Barriers to provision included stigma and lack of education. Commonly suggested strategies to improve practice included: increasing sexuality training, utilizing a team approach, initiating the conversation of sexuality early, and consensual inclusion of significant others in sexuality support. The results therefore indicate sexuality support is not routinely provided to people with SCI and findings suggest a need for sexuality training, utilizing a team approach, initiating the conversation, and including significant others.

This study sought to understand what sexuality support Australian health professionals currently provide to people with spinal cord injury (SCI) and their perspectives on what changes may better support the sexuality needs of people with SCI. Australian Health professionals who had worked with people with SCI within the last 10 years were invited to participate in an online survey. Results were analyzed using content analysis and descriptive statistics. The 39 participants were from a range of health professions including medical, allied health, nursing, and peer support. Participants worked in various service settings, with the highest frequency in the community (33%) or inpatient rehabilitation (28%). Analysis indicated 85% of participants had provided sexuality support, however this provision was rarely routine. Discussing sexuality education topics were reported to be routinely provided for less than 16% of participants. Overall, 32% of participants felt sexuality was addressed ‘not well at all’ in their workplace. Qualitative analysis of open-ended responses produced five themes: *barriers to supporting sexuality, health professionals require training, utilizing a team approach, responsibility to initiate conversation*, and *involving others in support.* Barriers to provision included stigma and lack of education. Commonly suggested strategies to improve practice included: increasing sexuality training, utilizing a team approach, initiating the conversation of sexuality early, and consensual inclusion of significant others in sexuality support. The results therefore indicate sexuality support is not routinely provided to people with SCI and findings suggest a need for sexuality training, utilizing a team approach, initiating the conversation, and including significant others.

## Introduction

There is increasing attention in healthcare research on sexuality after spinal cord injury (SCI) [1]. However, sex-related topics are often avoided in healthcare, particularly in the context of disability and as such, are often neglected by healthcare professionals (HCP) [2]. Sexuality support has traditionally been outcome-oriented and medically driven, with a focus on fertility, erections, and/or ejaculation [1, 3]. Qualitative research, however, points to the need for a broader focus, with people with SCI reporting that HCP undervalue the importance and scope of sexuality, and that the resources they receive are provided are outdated and/or limited [4].

According to the World Health Organization (WHO) [5], sexuality, which encompasses sex and intimacy, is a central aspect of being human and is closely interlinked with concepts of self-esteem and quality of life [6]. When the sexuality needs of people with SCI are not met, they can experience poorer physical, emotional and psychosocial outcomes [7]. Historically, society has tended to consider people with disabilities as either non-sexual or unable to have sex [8]. However, reconceptualizations of sexuality and disability are developing (albeit slowly) as normative/ableist perspectives are increasingly challenged, enabling people with disabilities to express their sexuality more freely [8], and providing avenues for HCP to rethink their approaches. To ensure HCP are providing comprehensive sexuality support in line with contemporary conceptualizations when working with people with SCI, enhanced education, knowledge and exposure to concepts of sexuality and disability are required [8]. In line with contemporary definitions of sexuality, sexuality support in this paper is defined as the healthcare management of sexuality related concerns after SCI.

Assessing a person’s sexual needs and providing sexual education and counselling facilitates provision of comprehensive/appropriate sexuality support [7]. Additionally, having a multidisciplinary or interdisciplinary rehabilitation team available to provide sexuality support soon after the injury is recommended [7, 9–11]. This was highlighted in an Australian study, where a sexuality training program for multidisciplinary SCI rehabilitation teams had immediate and long-lasting positive results on practitioner knowledge, comfort and attitudes when addressing sexuality issues with clients [12, 13]. Guidelines also exist which detail how sexuality, sexual health and reproductive health can be supported for adults with spinal cord injuries [14, 15]. Despite this existing research which suggests potential benefits of providing comprehensive support for sexuality after SCI, to the authors’ knowledge, is not well understood what type of support is currently being provided within Australia or where gaps exist. This study sought to address this concern, posing the following research questions:

- What support for people’s sexuality after SCI is/has been provided in a variety of service settings in Australia?
- From the perspectives of HCP, what service improvements (if any) can better support people’s sexuality after SCI in Australia.

## Methods

### Study design

This study used a cross-sectional survey methodology to obtain both quantitative and qualitative data.

### Participants and recruitment

Participant recruitment involved purposeful, convenience and snowball sampling methods. An initial call for interest was via advertising on social media platforms, direct messaging to HCP working with people with SCI (identified by online professional profiles), emailing researchers’ existing networks, and contacting relevant organizations via phone or email. The initial call provided a brief study overview with a link to the custom-made survey containing information about the study, eligibility, and a requirement to consent before commencing. HCP were eligible to participate if they had worked in Australia with people with SCI within 10 years. Survey responses were included if they were at least 80% complete.

As this study did not seek to find statistical significance, as per the analysis section below, the authors did not seek to include a minimum sample size. However, the length of time the survey was open was extended to maximize the number of participants. This was especially important considering the extra burden placed on HCP at this time due to the onset of the Covid-19. Within Australia, there are ten specialist SCI services [16], and most states have a major community SCI service. Each major service employing a varying number of HCP from most health disciplines. Some states of Australia have SCI units as well as outreach services. The participants recruited were from a range of service settings and all states were represented in this sample.

### Data collection

The 57 question survey was created using Qualtrics© [17]. The survey included 47 closed questions (Likert scales and multiple-choice) on topics including: interventions provided, disciplinary scope, training, and how conversations are initiated. Ten open-ended questions were also included and focused on, suggestions to improve practice, existing barriers, involving significant others, and sexuality support timing. The custom-made survey (see supplementary material) was created based on previous literature and in consultation with two consumers and health professionals. Three HCP who work in SCI units, including one physician and two occupational therapists, then pilot tested the survey. The survey was revised and opened from March 2020 to January 2021. Completed survey data were exported into Microsoft Excel (2008) for analysis.

### Analysis

Descriptive statistics were used for quantitative data. For qualitative data, manifest content analysis was used as described by Bengtsson [18]. The analysis involved coding, categorizing and theming the data based on four stages: decontextualization, recontextualization, categorization and compilation [18]. To enhance rigor, initial codes and themes were created by CB and these were then reviewed and revised by TA and JS until consensus was achieved.

## Results

A total of 39 HCP participated in the study including: allied health (*n* = 26), nurses (*n* = 4), peer support workers (*n* = 4), doctors (*n* = 3), and sexuality professionals (n = 2). All Australian states were represented. Most participants worked in community (33%, *n* = 13) and inpatient rehabilitation settings (28%, *n* = 11). Participants were mainly female (77%, *n* = 30), aged 31–45 years (59%, *n* = 23) and had been working with people with SCI for 1–5 years (39%, *n* = 15). See Table 1.

**Table 1:** Demographic characteristics

Category	n (%)
**Age (Years)**	
18–30	6 (15)
31–45	23 (59)
46–60	7 (18)
61–75	3 (8)
**Gender**	
Female	30 (77)
Male	8 (21)
No response provided	1 (3)
**Occupation**	
Doctor	3 (8)
Exercise physiologist	4 (10)
Leisure therapist	1 (3)
Nurse	4 (10)
Occupational therapist	9 (23)
Peer support worker	4 (10)
Physiotherapist	8 (21)
Psychologist	3 (8)
Sexuality professional	2 (5)
Social worker	1 (3)
**Time working with people with SCI**	
1–5 years	15 (39)
5–10 years	7 (18)
10–20 years	12 (31)
20 + years	5 (13)
**States HCP work/ed with people with SCI**	
New South Wales	15 (32)
Queensland	11 (23)
South Australia	3 (6)
Tasmania	1 (2)
Victoria	12 (26)
Western Australia	5 (11)
**Most recent service setting HCP worked with people with SCI**	
Acute	2 (5)
Community	13 (33)
Inpatient rehabilitation	11 (28)
Outpatient rehabilitation	3 (8)
Private practice	8 (21)
Transition care	2 (5)

Quantitative results are presented first under the topic headings: *overview of sexuality support, provision of interventions and management strategies, sexual education and information, sexuality and SCI training and initiation of conversation*. Qualitative results are reported separately in the subsequent section.

### Quantitative results

#### Overview of sexuality support

Participants felt sexuality was addressed slightly well (35%, *n* = 13) or not well at all (32%, *n* = 12) in their workplace, with only 22% (*n* = 8) selecting moderately well and 11% (*n* = 4) very well. Of the 39 participants, 85% (*n* = 33) had provided sexuality support to people with SCI. Participants who reported never providing sexuality support were exercise physiologists (50%, *n* = 2), physiotherapists (38%, *n* = 3), and occupational therapists (11%, *n* = 1). See Table 2 for more detail.

**Table 2:** Participants who had provided sexuality support

Health professional	n (%)
Doctor	3 (100)
Nurse	4 (100)
Occupational therapist	8 (89)
Peer support worker	4 (100)
Psychologist	3 (100)
Physiotherapist	5 (63)
Sexuality professional	2 (100)
Social worker	1 (100)
Leisure therapist	1 (100)
Exercise physiologist	2 (50)
Total	33 (85)

Participants reported that in their service settings, the HCP that most frequently provided sexuality support were doctors (64%, *n* = 25) and nurses (64%, *n* = 25). The service settings with the highest frequency of sexuality support provided by these HCP were inpatient rehabilitation (100%, *n* = 11), outpatient rehabilitation (100%, *n* = 3), and transition care (100%, *n* = 2). Table 3 provides further information about participants’ perspectives of which HCP provide sexuality support in their service setting.

**Table 3:** Participants perspective on which HCP provides sexuality support within their service setting

HCP	Service setting *n* (%)						
	Acute *n* = 2 (5.1%)	Inpatient rehabilitation *n* = 11 (28.2%)	Outpatient rehabilitation *n* = 3 (7.7%)	Transition care *n* = 2 (5.1%)	Community *n* = 13 (33.3%)	Private practice *n* = 8 (20.5%)	Across all settings *n* = 39 (100%)
Doctor	1 (50)	11 (100)	3 (100)	2 (100)	6 (46)	2 (25)	25 (64)
Nurse	1 (50)	11 (100)	3 (100)	2 (100)	6 (46)	2 (25)	25 (64)
Occupational therapist	1 (50)	6 (55)	0 (0)	1 (50)	7 (54)	3 (38)	18 (46)
Peer support worker	1 (50)	5 (45)	2 (67)	0 (0)	7 (54)	2 (25)	17 (44)
Psychologist	1 (50)	5 (45)	2 (67)	1 (50)	6 (46)	1 (13)	16 (41)
Physiotherapist	1 (50)	4 (36)	0 (0)	1 (50)	6 (46)	1 (13)	13 (33)
Sexuality professional	1 (50)	3 (27)	1 (33)	0 (0)	5 (38)	1 (13)	11 (28)
Social worker	1 (50)	4 (36)	0 (0)	1 (50)	4 (31)	1 (13)	11 (28)
Leisure therapist	0 (0)	2 (18)	0 (0)	0 (0)	5 (38)	0 (0)	7 (18)
Exercise physiologist	0 (0)	0 (0)	0 (0)	0 (0)	2 (15)	0 (0)	2 (5)

#### Provision of interventions and management strategies

Interventions and strategies participants reported providing are shown in Table 4, with those most frequently provided including ‘information and education on sexuality’ (74%, *n* = 29), ‘referring on to alternative health professional/service’ (56%, *n* = 22), ‘delivery of a workshop or program’ (28%, *n* = 11) and ‘sexual counselling’ (28%, *n* = 11).

**Table 4:** Sexuality management strategies previously provided by participants

Intervention	n (%)
Provision of information/education about sexuality	29 (74)
Referral/recommendation to another health professional/service	22 (56)
Delivery of a workshop or program which addresses sexuality	11 (28)
Sexual counselling	11 (28)
Information/recommendation to services from a sex worker	8 (21)
Medications to support sexual function	7 (18)
Assessments related to sexuality	7 (18)
Peer support sessions	6 (15)
Fertility advice and/or treatments	6 (15)
Recommendation or prescription of alternative or complimentary medicine/treatments	4 (10)
Other - “general talks with clients”	1 (3)

**Fig. 1:**
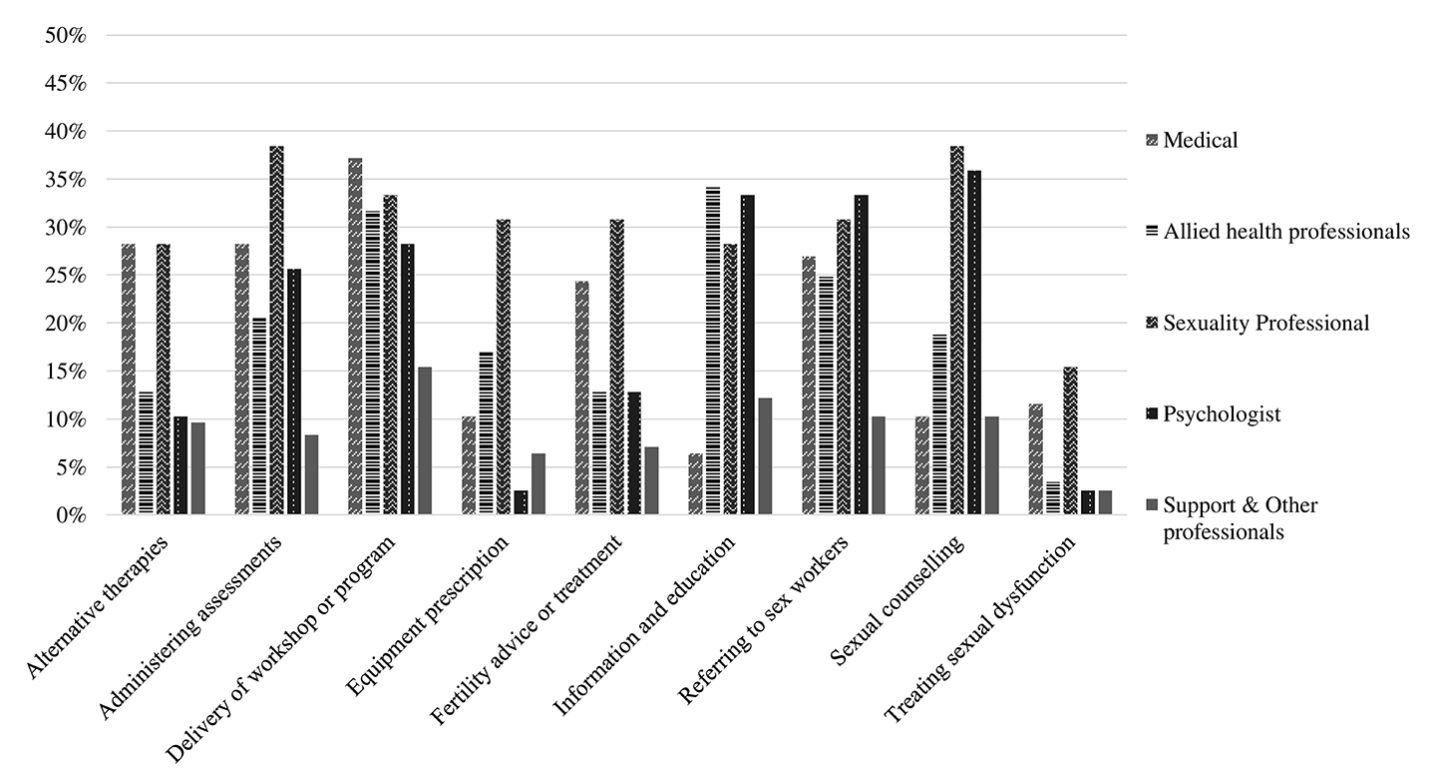
**Percent HCP who should be providing additional sexuality support.** This figure depicts the % of HCP who should be utilizing various support strategies (in relation to those who already do) within the participants’ service setting

Participants also reported on the interventions and strategies provided by each HCP within their service setting. The most frequent interventions provided in their settings were for sexual dysfunction (67%, *n* = 26) and fertility (64%, *n* = 25), provided by doctors. Management strategies that participants most frequently reported were not being provided by any HCP within their service included: alternative therapies (72%, *n* = 28), assessments (54%, *n* = 21), and referring to sex workers (51%, *n* = 20). Participants also indicated that all HCP should be utilizing sexuality management strategies more frequently than current practice (Fig. 1).

#### Sexual education and information

**Fig. 2:**
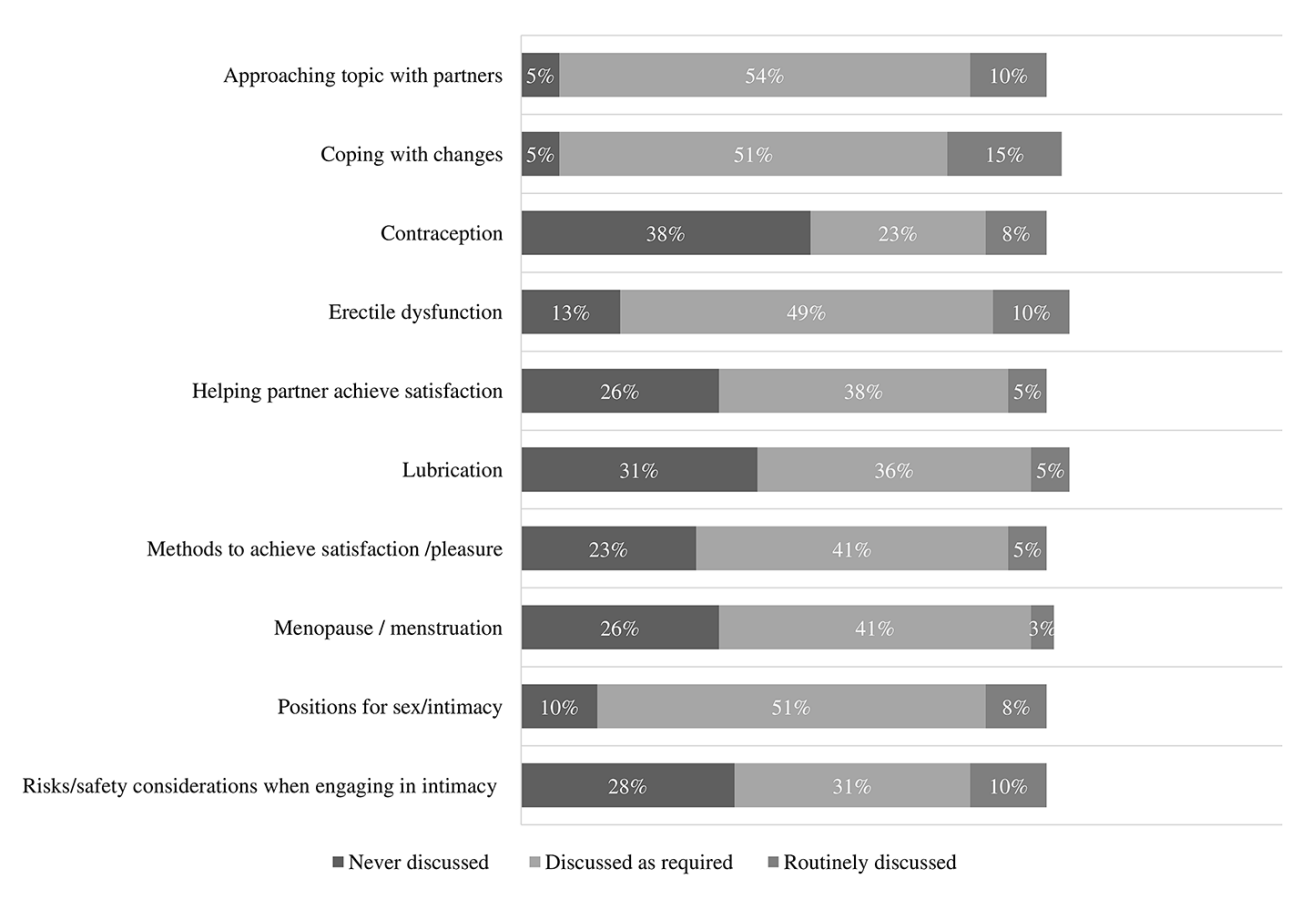
**Education and information topics.** Frequency that participants routinely provide various sexuality education and information topics

As discussed above, participants were provided sexual education most frequently and the service settings where participants were most frequently provided education were outpatient services (100%, *n* = 3), transition care (100%, *n* = 2) and inpatient rehabilitation (91%, *n* = 10). However, Fig. 2 shows that various education topics do not appear to be routinely discussed. For example, the topic of ‘coping with changes’ is the most routinely provided sexuality education topic, however, this is only routinely provided by 15% (*n* = 6) of participants. Topics that participants reported to discuss least were contraception, lubrication, and safety considerations.

**Fig. 3:**
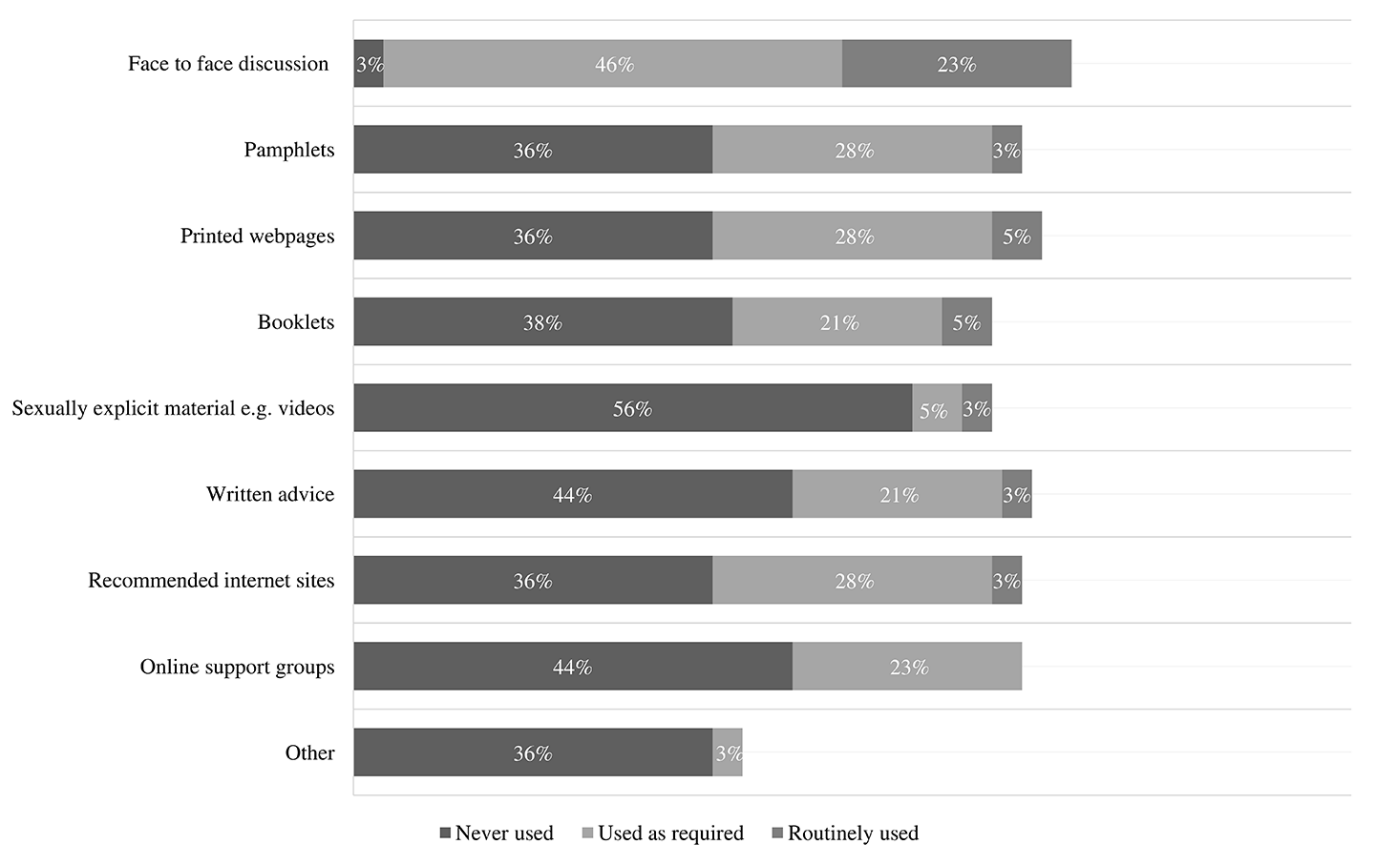
**Format of education provision**. Frequency that participants use various formats to provide sexuality education and information

How frequently participants report using various methods of providing sexual education is depicted in Fig. 3. Face-to-face discussions were routinely used by the greatest number of participants (23%, *n* = 9), with all other methods routinely used by less than 6% of participants. For example, only one (3%) participant routinely provided written information.

#### Sexuality and SCI training

Only 27% (*n* = 10) of the 37 participants who responded to the training questions reported having received general sexuality training, and 51% (*n* = 19) sexuality training specific to people with SCI. Almost all participants said they wanted more sexuality training: 87% (*n* = 32) general sexuality training and 97% (*n* = 36) sexuality training specific to people with SCI. Participants identified the following sexuality and SCI related training topics they wished to receive: information on resources and services (82%, *n* = 32), assistive equipment (74%, *n* = 29), resources about initiating the topic of sexuality (69%, *n* = 27), practical education sessions (69%, *n* = 27), reproductive and fertility information (67%, *n* = 26), administering assessments (62%, *n* = 24), recommending sex workers (56%, *n* = 22), basic information on sexuality after SCI (54%, *n* = 21), and group therapy provision (51%, *n* = 20).

#### Initiation of conversation

When the conversation of sexuality is initiated, 44% (*n* = 16) of participants reported that they initiate the conversation whilst 31% (*n* = 11) said patients initiate it. 25% (*n* = 9) of participants reported that initiation varies between the patient and themselves. When asked where the topic should be initiated, most participants reported that it should be initiated in inpatient rehabilitation (79%, *n* = 31), followed by acute care (38%, *n* = 15), outpatient (23%, *n* = 9), community (18%, *n* = 7), transition care (13%, *n* = 5), and private practice (15%, *n* = 6).

### Qualitative results

The qualitative results are reported under the following five themes: *barriers to supporting sexuality, health professionals require training, utilizing a team approach, responsibility to initiate conversation*, and *involving others in support.*

#### Barriers to supporting sexuality

Overwhelmingly participants said that sexuality remains an uncomfortable and stigmatized topic, particularly in the context of disability, and sexuality support is not consistently provided or prioritized in practice. Participants stated that sexuality *“is just not discussed openly*” (HP7, leisure therapist), and to decrease the stigma attached to sexuality, HCP need to have, “*more open discussions*” (HP11, physiotherapist).

As well as acting to encourage conversations around sexuality, participants suggested service settings should prioritize sexuality support as a routine part of healthcare. One participant suggested HCP need to start, “*identifying sexuality and fertility as a formal part of the rehabilitation program*” (HP31, occupational therapist). Participants discussed that sexuality is viewed as having little importance and an existing barrier is, “*the disconnect between what clients find important vs. what healthcare workers do*” (HP11, physiotherapist).

#### Health professionals require training

The strongest suggestion participants made to improve practice was to train HCP to provide better sexuality support with people with SCI. One participant suggested, “*more training to ensure everyone is confident and capable to initiate the conversation*” (HP21, Nurse). The type of training participants suggested included: continual learning opportunities, in-service discussions, and disseminating relevant research amongst the team. However, participants also said that services lack time and/or resources to provide training opportunities.

#### Utilizing a team approach

A dominant reoccurring message throughout the open-ended responses was that a team approach is vital when providing sexuality support. This is demonstrated by the following participant quotes: “[need] *more of a team approach*” (HP6, occupational therapist), “[need] *more disciplines involved*” (HP9, physiotherapist), “*so important to be multidisciplinary…it’s a team approach*” (HP29, exercise physiologist). Participants also noted that it would be highly beneficial to have a dedicated sexuality professional within the team. For example, a nurse stated,

*Having a Sexual Health Nurse Consultant service at our organization really means staff can open a conversation about sexuality and know there is a referral option with a health professional who has received additional training in this area* (HP21, nurse)

Utilizing a team approach and understanding team roles in addressing sexuality was said to assist HCP when referring. For example, one physiotherapist said, “[need] *more of a team approach and an understanding of each disciplines role so it is easy within the team to refer to each other”* (HP11).

#### Responsibility to initiate conversation

Participants expressed a need for HCP to assume responsibility for initiating conversations about sexuality with people with SCI. Reasons why the initiating party may vary included HCP waiting to get a sense of what the patient might want. For example, one participant stated, *“some patients bring it up, others tend to hint at wanting information but potentially feel awkward asking so I would start a general conversation”* (HP26, occupational therapist).

Many participants discussed that sexuality conversations should be initiated as early as possible. Some participants suggested inpatient rehabilitation and acute settings as examples of appropriate settings to initiate the conversation. However, participants also emphasized that the conversation should be raised at various time points across the continuum of care to ensure people have multiple opportunities to discuss the topic. For example, one participant explained why they took this approach, “*as some people are not ready to talk about it at certain times and are more engaged at later or earlier times*” (HP37, psychologist).

#### Involving others in support

Another prominent theme identified was that sexuality support should include anyone the person nominates. However, participants said that the topic of sexuality should not be initiated when others are present and only discussed with others after the patients have previously provided consent. One participant stated, “[it is] *important to have* [their] *partner there to discuss the issues if both give consent*” (HP30, nurse). Another participant also noted parents of children/adolescents with SCI may be important to include, especially when topics of fertility arise.

## Discussion

This study investigated what sexuality support HCP are providing to people after SCI. The results show sexuality support appears to be inadequate as the participants’ most frequently reported sexuality was only addressed ‘slightly well’ and ‘not well at all,’ the lowest two points on the four-point scale. As shown in Fig. 2, all education and information topics were never provided more than 15% of the time, indicating that the provision of basic sexual education and information, appears to not be routinely provided.

This apparent infrequent and inadequate support echoes the perspectives of people with SCI [4, 19–22]. For example, recent qualitative research investigating men’s’ experiences of sexuality support post SCI in Canada found inadequate support was received for sexual adjustment, such as HCP having poor knowledge and comfort when addressing sexuality [4].

Support for sexuality has previously largely focused on highly medicalized areas of sexuality, such as prescribing Viagra to achieve erections [1]. This study’s findings support this as the most frequently provided interventions were for medical treatments by doctors. However, our results indicate there is a demand for further input from the wider team, including sexuality professionals.

Although research points to the value of using of a range of sexuality interventions as part of a comprehensive approach [1], in the Australian context, it was found that few interventions are routinely provided in practice. Our findings also indicate, as depicted in Table 4, that some management strategies, such as ‘peer support sessions’ (15%) were less utilized than others, for example, sexual education (74%). This finding contrasts with evidence-based practice recommendations to administer sexuality assessments as an important first step when providing sexual rehabilitation [23–25]. Peer support sessions have also been found to be empowering and motivating for people with SCI [26]. The lack of utilization of these and other approaches suggest that comprehensive sexuality support is needed which routinely utilizes a wide variety of management strategies, such as administering sexuality assessments, peer support sessions focused on sexuality, sexual counselling, delivering workshops or programs for individuals and/or couples, and providing information about sex work services. Another important part of providing support is initiating the conversation on sexuality. The results of this study, and a recent scoping review [1], suggest that healthcare services should ensure HCP initiate the conversation multiple times across the continuum of care to capture a time when the person is ready to address sexuality.

While sexual education was the most provided sexuality intervention in this study (see Table 4), the most routinely provided education topic was only reported to be routinely provided by 15% of participants (see Fig. 2). Low levels of educational interventions have also been reported in the USA, with less than 50% of people with SCI receiving sexual education and counselling during rehabilitation in a 1992 study [27]. More recent studies, published in 2019 and 2020 [28, 29], indicate that provision of sexual education continues to be limited. Taken together, this work spanning three decades demonstrates the persistent challenges of improving sexuality support for people with SCI.

Limited variation in education methods were also reported in this current study with HCP tending to rely on face-to-face conversations. One of the least used methods was videos, yet the use of sexually explicit films/videos to provide sexual education/information has been considered helpful in removing mysteries around sex for people with SCI [30]. This lack of variation limits the possibilities for versatile, person-centered care. As person-centered care is considered key to quality healthcare, it is important to find multiple/flexible ways to approach sexuality support.

Our study indicates some of the barriers to sexuality support after SCI and this understanding might help focus efforts to address the inadequacy of sexuality support currently provided in the Australian context. Stigma around sexuality emerged from the qualitative findings as one of the largest barriers. Although there have been significant changes in Australian societal attitudes in recent decades, seen for example in the legalization of same-sex marriage in Australia in December 2017 [31]. attitudes towards sexuality continue to be somewhat conservative, particularly for people with disabilities [32]. This issue is not isolated to Australia, in a recent Canadian Delphi survey, the participating men with SCI indicated that society appears to have misconceptions regarding a person’s sexuality after SCI [33]. Arguably, despite apparent wider social acceptance and discussions of sexuality, views on sexuality and disability may be slower to change in society and in healthcare practice. Increasing education and awareness within society and healthcare may help to improve the stigma attached to sexuality after SCI.

In addition to stigma, another barrier to providing comprehensive sexuality support for people with SCI identified in the study was a lack of HCP education. Training HCP about sexuality after SCI has been found to increase their comfort and knowledge around providing sexuality support, including their ability to initiate sexuality conversations [12, 13]. Guidelines are another resource which HCPs can refer to for further education [14, 15]. Guidelines and training are important tools when reviewing or developing sexuality support services with people with SCI. However, further research which investigates optimal ways to educate and train HCP on sexuality after SCI is likely to be valuable.

Finally, this study indicated the importance of involving others in sexuality support, however, HCP should first obtain consent. In related qualitative research, intimate partners of people with SCI reported that they desired more sexual support from HCP as support was either unavailable to them or too general [34]. These findings suggest that in practice, sexuality support should include partners and/or other important people when discussions of sexuality arise and where consent has been provided from the individual first.

### Limitations

Given the small number of professionals working in the field nationally, a relatively small sample size with a limited number of each discipline represented was anticipated. However, the results should be considered with the sample size in mind as well as the context of the study, where results may not be transferable across countries/populations. Additionally, this study used convenience sampling methods to recruit participants and future research may benefit from a larger sample size and probability sampling methods.

#### Future research

This study focused on the perspectives of HCP, future research should aim to understand the perspectives of a range of stakeholders on support received, particularly people with SCI and/or intimate partners. This study has also highlighted the importance of providing training for HCP. Further research exploring the specific training needs of HCP and the development of training would be beneficial.

## Conclusions

HCP working with people with SCI in Australia consider sexuality to be an important part of healthcare. However, while this is a convenience sample, the results of this study suggest sexuality support is not routinely provided in Australia and suggests limited variety in the support provided. Despite wider acceptance of the diversity of sexuality in society, disability and sexuality in healthcare appear to continue to be stigmatized and sidelined. Along with stigma, a lack of education amongst HCP on sexuality after SCI is a barrier to sexuality support. To improve the sexuality support provided after SCI, recommendations for practice include: utilizing a range of sexuality management strategies using various delivery methods to help increase person-centered care, utilizing a team approach which extends beyond the traditional medical approach to sexuality, providing training to HCP, encouraging more open discussions regarding sexuality to decrease stigma, HCP initiating the conversation about sexuality at multiple points across the continuum of care, and HCP including others in the sexuality support provided, when consent is obtained.

## Data Archiving

The datasets analyzed during the current study are available from the corresponding author on reasonable request.
